# Perceptions of livestock value chain actors (VCAs) on the risk of acquiring zoonotic diseases from their livestock in the central dry zone of Myanmar

**DOI:** 10.1186/s12889-022-14968-y

**Published:** 2023-01-30

**Authors:** Tu Tu Zaw Win, Angus Campbell, Ricardo J. Soares Magalhaes, Kyaw Naing Oo, Joerg Henning

**Affiliations:** 1 The School of Veterinary Science, The University of Queensland, Gatton, Australia; 2 Present Address: Centre for Applied One Health Research and Policy Advice, City University of Hong Kong, Hong Kong, 999077 Hong Kong SAR, China; 3 Faculty of Veterinary & Agricultural Sciences, The University of Melbourne, Melbourne, Australia; 4 UQ Spatial Epidemiology Laboratory, The School of Veterinary Science, The University of Queensland, Gatton, Australia; 5 Children’s Health and Environment Program, The University of Queensland, The University of Queensland, South Brisbane, QLD 4101 Australia; 6 Livestock Breeding and Veterinary Department, The Ministry of Agriculture, Livestock and Irrigation, Nay Pyi Taw, Myanmar

**Keywords:** Zoonoses, Health belief model, Livestock farmers, Value chain actors

## Abstract

**Objectives:**

The Central Dry Zone (CDZ) is one of the most important livestock production areas of Myanmar. However, there is an eminent lack of information on the attitudes and traditional beliefs of local farmers and livestock supply chain actors in CDZ of Myanmar on the public health implications. A modified data collection instrument of the Health Belief model was developed to investigate attitudes, beliefs and barriers to the application of recommended zoonotic disease prevention.

**Study design:**

Cross-sectional study.

**Method:**

Data analyses were conducted considering a two-phase multilevel mixed effect binomial generalized linear models modelling approach.

**Results:**

The availability of information about zoonosis to supply chain actors influenced their confidence to implement preventive actions (OR = 1.5, *p* = 0.045 for cattle diseases; OR = 1.5, *p* = 0.022 for village chicken diseases). Supply chain actors were more likely aware of zoonosis transmitted by cattle compared to livestock farmers (OR = 0.3, *p* = 0.005 for cattle farmers), while people not rearing or trading small ruminants and/or poultry were less likely to be aware of the zoonotic risk associated with these animals (*p* < 0.005). Information on zoonosis transmitted from small ruminants was mainly promoted through farmers (*p* = 0.032), while information on zoonotic diseases that can be obtained from chickens was disseminated through farmers, local authorities and the media. Nevertheless, appropriate hand hygiene measures (i.e. cleaning of hands after touching, cutting, cooking meat) (OR = 7.7, *p* < 0.001 for zoonotic small ruminant diseases; OR = 1.6, *p* = 0.073 for zoonotic village chicken diseases) and treating of sick animals (OR = 7.3, *p* < 0.001 for small ruminant zoonotic diseases; OR = 2.2, *p* = 0.031 for village chicken zoonotic diseases) increased the confidence of small ruminant and village chicken owners to prevent these zoonotic infections.

**Conclusions:**

The findings from this study indicate that while gender and the availability of information on zoonotic risks play an important role on the perceived threat of zoonoses, the practice of prevention methods influenced the confidence of value chain actors (VCAs) on zoonoses prevention.

## Introduction

Approximately 60% of all human infectious diseases originate from animals [1–3]. Zoonotic diseases such as anthrax, brucellosis, rabies, Japanese encephalitis, Q fever, *Trichinella* spp., tuberculosis, salmonellosis and avian influenza are significant threats to global population by affecting general population health, food security, food safety, economic and social development [4].

Zoonotic infection has been threatening the world population with wide spread geographical distribution. Due to its negative impact, zoonoses remain a public health challenge in the regions with limited resources [5]. The population of Myanmar has experienced a number of zoonotic disease outbreaks including anthrax [6–8], brucellosis [9, 10], highly pathogenic avian influenza (HPAI) and avian salmonellosis [9, 10]. As in many developing nations with limited veterinary services and poor health management, zoonotic parasitic infections [11, 12] including ascariasis, coccidiosis, fascioliasis, oesophagostomiasis, haemonchus infection, strongyloid nematode infection, have been reported in Myanmar [9, 10, 12]. These reports reflect the potential for zoonoses sharing between animals and humans within the region, and it could be the major threat to local farmers and livestock supply chain actors, who work closely with animals. However, the knowledge and perception of local farmers and supply chain actors on the risk of zoonotic diseases has not been widely observed.

A number of factors promoting human-animal interactions and triggering the introduction of zoonoses includes social and traditional behaviours (e.g. food habit, lack of adequate health care, and farming practice, living close to animals), demographic factors (e.g. sex, age, experience), environmental factors (e.g. global climate changes), pathogenic factors (e.g. genetic changes in pathogens) [13–17] and management factors (e.g. poor sanitary regulations, poor health management and inadequate veterinary services) [18–22]. Furthermore, a lack of knowledge on disease prevention methods, poor biosecurity practices and diseases dynamics are a matter of concern to developing countries [5, 23]. Therefore, it is crucial to raise the awareness of zoonotic threats, and thereby promote the self-efficacy of farmers and supply chain actors (SCAs) on zoonotic disease prevention (i.e. ability to prevent the zoonotic diseases being transmitted from livestock species to humans). Additionally, animal movement has been notorious for being one of the important factors favouring the spread of both livestock and zoonotic diseases [24, 25] which may further lead to cause public health problems. This raises our interest in exploring the perception of stakeholders (i.e. farmers and supply chain actors) on zoonoses and their prevention practices. The communication and knowledge sharing among different levels of stakeholders in trade routes might promote the accessibility to zoonoses information and this might compound awareness of zoonoses threats.

To improve the control of zoonoses by livestock value chain actors (VCAs) or stakeholders in the CDZ, we need to understand the limitations and opportunities for improving the attitude and practice of these stakeholders relating to the threat of zoonoses. The Health Belief model was firstly introduced to the health educational research in the 1950s by social psychologists Hochbaum, Rosenstock, and Kegels, who worked with the U.S. Public Health Service [26, 27] to look at the relationship between human cognitive behaviour, and practice of health preventive measures. It has been widely used among health psychology researchers. The Health Belief framework has been successfully used in determination of the psychological influence on taking preventive action in many human health researches [28–30]. However, the use of the Health Belief framework for disease prevention practice has not been widely seen in veterinary medicine.

The aim of this study is to assess the zoonoses belief and practice among selected livestock value chain actors (VCAs) in the Central Dry Zone (CDZ) of Myanmar in the CDZ. This will help to support the development of strategies to overcome constraints on zoonoses control and promoting the health status of VCAs in the CDZ of Myanmar under the one-health paradigm.

## Methods

### Study design

A cross-sectional questionnaire survey was conducted among small-scale farming households owning different livestock species in two administrative areas (townships), Myingyan and Meikhtila, in the CDZ of Myanmar. These two CDZ townships were key research sites for a larger livestock project (DAHAT PAN project), funded by the Australian Centre for International Agriculture Research (ACIAR), and been previously identified as representative of livestock production systems and practices performed throughout the wider CDZ [31].

### Sample size calculation and selection of sampling units

#### Farmers

For the selection of farmers, a two-stage sampling approach was used to identify villages and households in the survey, with primary sampling units (PSU) being villages and secondary sampling units (SSU) being households. Sample size calculation was done by using Epi Tools [32]. The proportion of farm income generated from livestock production was used as the outcome of interest for the sample size calculations, conservatively assumed to be 50%, with within- and between-cluster variances of ±10 and ± 2.5%, respectively. The low between-cluster variance reflected very similar ecological conditions resulting in similar income generation from livestock production across villages in the CDZ. Assuming that the proportion of farmers in a village deriving at least half of their income from livestock production was 0.7, a population of 400 villages per township and approximately 200 households per village (based on livestock statistics data compiled (LBVD 2014)), a precision of the estimate of ±5% with a 95% confidence interval, the estimated sample size was 20 households per village and 38 villages across the two townships. Lists of villages were provided by Livestock Breeding and Veterinary Department (LBVD), Myanmar. In order to select villages, a probability-proportional-to-size sampling strategy was used (http://epitools.ausvet.com.au/content.php?page=2StagePrevalence1), giving larger villages a greater probability of being selected. A total of 40 villages were selected in each township (20 villages to be selected and 20 potential replacement villages). Within selected villages, lists of households for each of the three major livestock species (cattle, small ruminants and village chickens) were provided by village headmen. Selected villages were replaced if they had insufficient households with the three livestock species of interest or if farmers were not willing to participate in the study. Overall, seven households from each livestock ownership list were randomly selected, providing a total of 21 households per village. Sample size calculations and random sampling were performed using the Survey Toolbox modules Sample size for 2-stage prevalence survey, Random sampling from a sampling frame (http://epitools.ausvet.com.au/content.php?page=RandomSampling1) and Random sampling of animals, respectively (http://epitools.ausvet.com.au/content.php?page=RandomSampling2). A total of 20 cattle farmers, 45 small ruminant farmers, and 54 village chicken farmers refused to participate in the survey and replacement households were randomly selected from the sampling frame. According to the calculations, we collected data from 21 livestock household in each of 40 villages, which lead to collect 280 households per species. Due of the overlapping among the different livestock ownership households, the data were collected from 328 cattle raising households, 303 small-ruminant raising households, and 327 village chicken raising households.

#### Supply chain actors

Stakeholders involved in livestock marketing network were identified using various approaches: a) they were identified by farmers in the household survey by specifying the trader’s phone number or/and living locations, b) they were identified on livestock markets and c) they were identified by asking interviewed supply chain actors about other supply chain actors they are knowing. The following marking locations were visited: two cattle markets, three bazaars, 10 village markets and 28 households where traders and middlemen were trading. Stakeholders involved in livestock marketing network were classified as follows:

- Middlemen: These are people involved in the trading network, who buy livestock (i.e. cattle or small ruminants or village chickens) from the farmers and sell them to traders or main collectors.
- Branch collectors: These are people involved in the trading network, who purchase livestock in the villages with the money provided to them by the main collector/traders. The branch collectors are employees of the main collectors.
- Main collector/Traders: These are people involved in the trading network, who buy the livestock from the middlemen or who employ the branch collectors. This group of people keep and trade a large number of animals and invest a large amount of money to set up the trading hubs.
- Hawkers: These people are selling goods, typically advertising them by shouting. They sell livestock products such as meat (not live animals), vegetables and food in the markets or in villages, to which they travel by motorbike or bicycle.
- Slaughterman: The people who hold license for slaughtering animals and also own abattoir. They usually collect livestock for slaughter from farmers, middlemen, branch collectors, and main collectors.

**Fig. 1:**
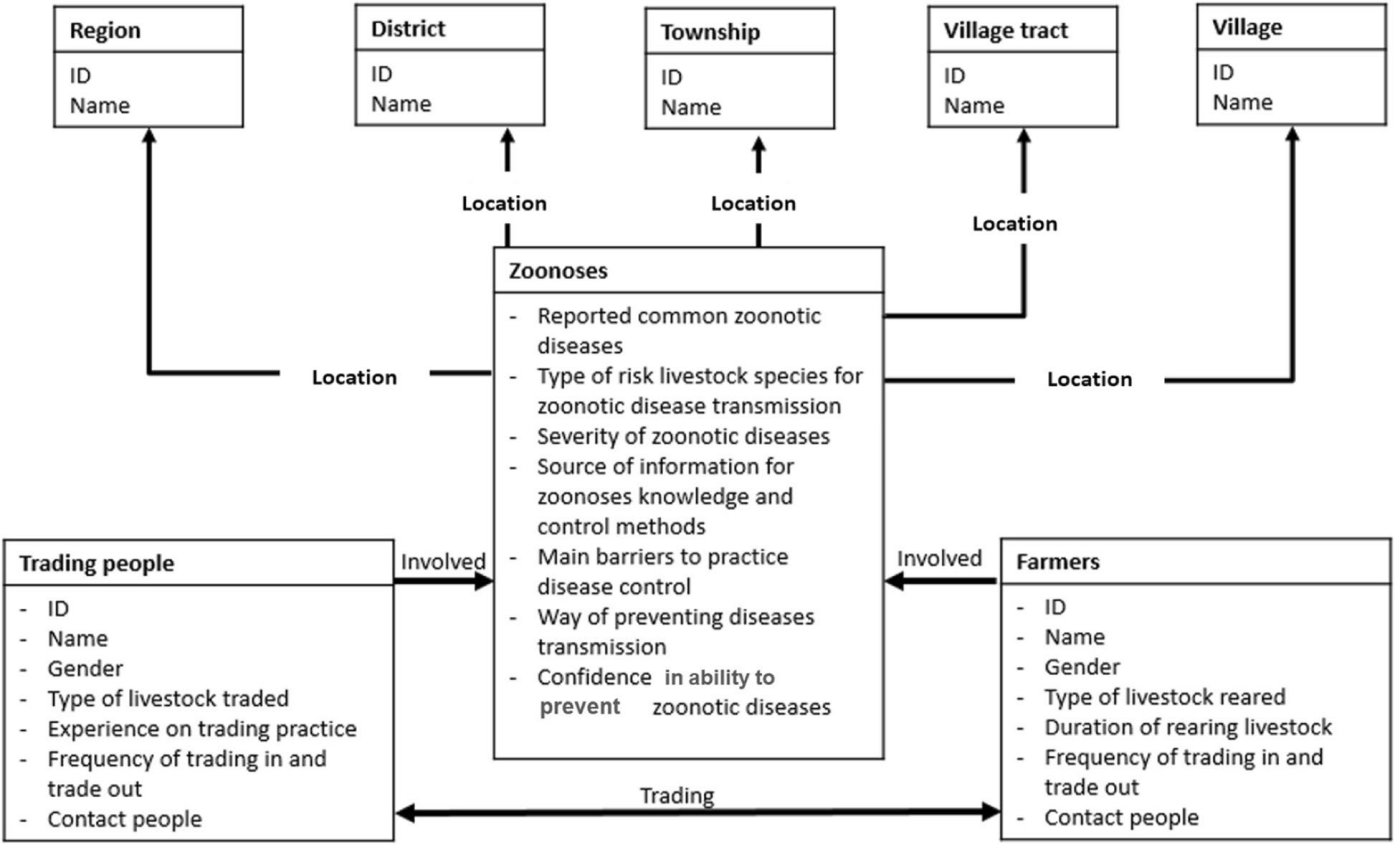
Data collection to understand the factors affecting the zoonoses control by VCAs

Data were collected from the different stakeholder groups involved in the livestock marketing network (i.e. farmers, hawkers, middlemen, branch collectors, and to describe the cross-species marketing network originating from small-scale livestock households in villages of the CDZ of Myanmar. Data collection were conducted over 1–2 days in each market location. Data were collected from all the main livestock supply chain actors (especially for small ruminants and village chickens) identified by farmers, livestock market managers, local veterinary authorities and members of local livestock federations whereas convenient sampling was undertaken with other supply chain actors (i.e. hawkers, middlemen, branch collector) in that locality. Interviews were conducted with a total of 31 middlemen, 19 traders, 11 hawkers, 1 cattle market managers, and 1 slaughterman. In data analyses, all levels of people mainly involved in trading including traders, middlemen, branch collectors, hawkers, and slaughtermen were categorised into one group, named “supply chain actors”. In this study, we named all the levels of stakeholders including both “farmers” and “supply chain actors” as “value chain actors (VCAs)”. The questions in the questionnaire were constructed by means of Health Belief modelling framework (Fig. 1).

### Data collection

#### Questionnaire survey

Questionnaires were developed in the English language. The questionnaire contained the following sections: demographic information, and perceptions on the impact of animal production on human health, and public health implications. The questions in the questionnaire were constructed by means of Health Belief modelling framework (Table 1).

**Table 1:** Health Belief Model on the impact of rearing different types of animals on human health

No.	Concept	Definition
1	Perceived Threat	Humans can become infected with disease from the relevant species (cattle, small ruminants or poultry).
2	Perceived Sever	The consequences of getting the disease from the relevant species (cattle, small ruminants or poultry) are significant enough to try to avoid for the benefit of human health.
3	Perceived Benefits	Recommended and proper husbandry system with biosecurity system can prevent the disease transmission from the relevant species (cattle, small ruminants or poultry) to humans.
4	Perceived Barriers	The barriers in practising proper biosecurity system and disease transmission between the relevant species (cattle, small ruminants or poultry) and humans.
5	Cues to Action	The main action that encourages VCAs to be aware of the zoonotic diseases transmitted from the relevant species (cattle, small ruminants or poultry).
6	Self-Efficacy	The farmers have confidence in knowing how to protect themselves from zoonotic disease from the relevant species (cattle, small ruminants or poultry).

According to the framework, data were collected on demographic information, livestock trade information, perception of farmers on risk of zoonoses from livestock species, the availability of information on risk of zoonoses, preventive actions, main barriers to disease prevention, and the level of confidence on zoonoses control (Fig. 1). Pilot testing of the questionnaire was conducted in three households within two villages in Meikhtila Township. The selection of these villages was conducted by analysing the score on wealth and development (1 = very poor, 2 = poor, 3 = moderate, 4 = good, 5 = very good). Scoring of the villages in Meikhtila Township was conducted by seven members of the local authority, three animal health workers and two junior scientists. Based on this ranking, one village with the highest score and one village with lowest score were chosen. In each village, three households with cattle production, sheep or goat production, and village chicken production, were surveyed. From the trading survey, the pilot test was conducted with three local traders in Bago region. After the pilot testing, a total number of six questions were modified. Questions on attitude, and practices to prevent transmissible zoonoses from livestock were adjusted and modified to be more relevant to the local conditions and improved to ensure that interviewees better understood the questions asked. The open-ended question for both farmers and supply chain actors (SCAs) were used to explore their perception and disease prevention practice without any clue. The perceptions and disease prevention practices described in this research were based on the report of VCAs.

After the questionnaire was finalized, a survey team was organized by seven enumerators. Enumerators were two students from the University of Yezin, four staff from LBVD and the author of this paper. Team members were trained in interviewing techniques and they familiarized themselves with the questionnaire before the survey commenced. Questionnaire interview was conducted with both supply chain actors groups and farmer groups. The duration of each interview was approximately 20 minutes.

### Data analysis framework

#### Conceptual framework for the analysis

**Fig. 2:**
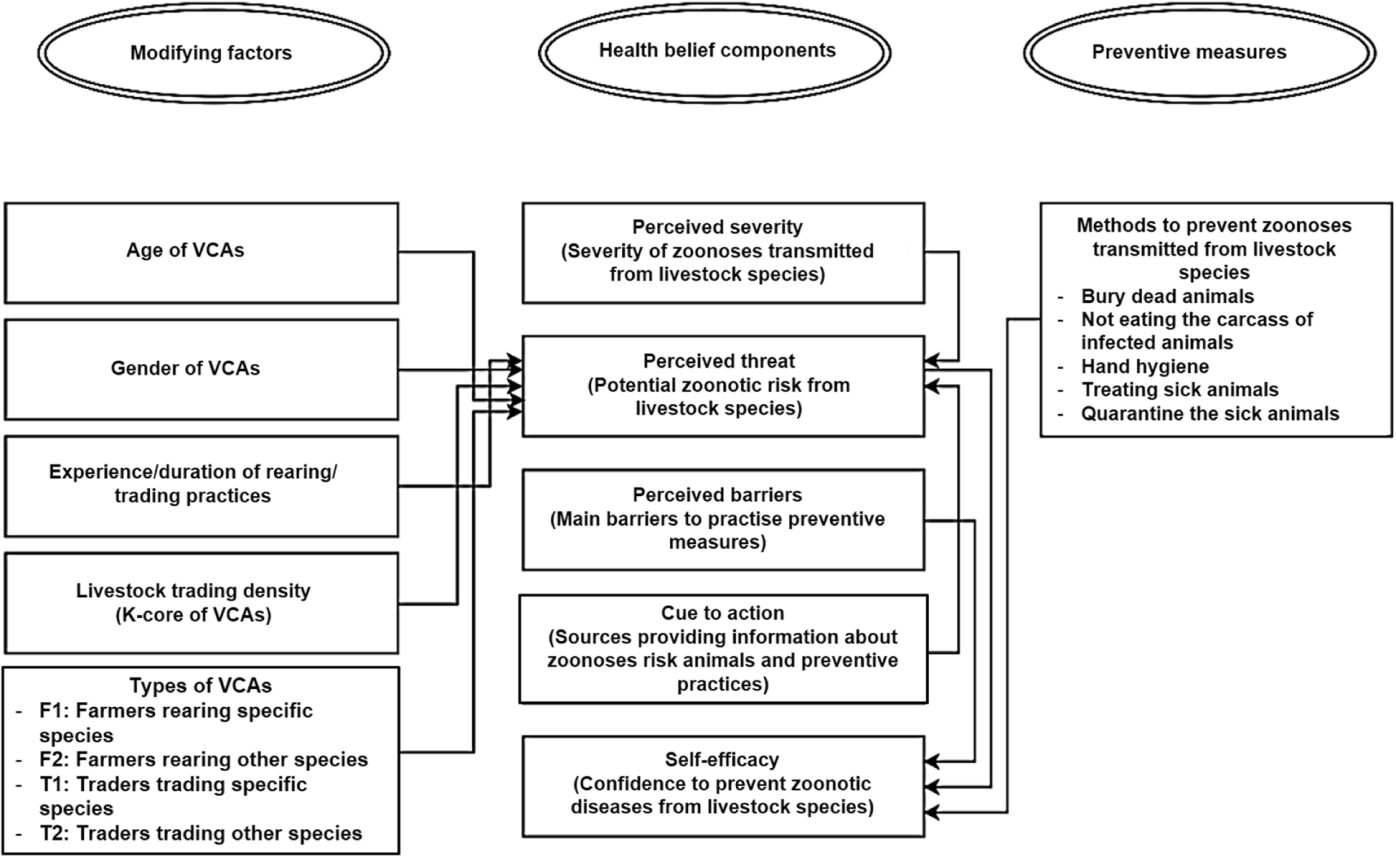
Causal diagram for health belief modelling framework on perception of zoonotic diseases by value chain actors (VCAs)

We adapted the Health Belief Model (HBM) to collected information on the health-belief components, such as perceived threat, perceived severity, perceived benefit, perceived barrier, cue to action and self-efficacy of farmers and supply chain actors towards the control of zoonotic diseases [33]. Our analyses were conducted in two phases: firstly, to understand the factors affecting any perceived threat and secondly, to understand the factors affecting self-efficacy of farmers on zoonoses control across different livestock species. To fulfil these objectives, we developed two interlinked models, one to model perceived threats of zoonoses and another to model self-efficacy (Fig. 2). In the first model, we assumed that awareness of potential zoonotic risk from livestock species (i.e. perceived threat) to be influenced by modifying factors (i.e. age, gender, experience in livestock rearing/trading, livestock trading density, type of career), information availability (i.e. cue to action) and awareness of VCAs on severity of transmissible zoonotic disease from livestock. Furthermore, in the second model, we assumed that self-efficacy (i.e. confidence in disease prevention) was influenced by awareness of the potential zoonotic risk from livestock species, disease prevention practices and barriers to practising disease prevention. In this study, we observed the disease prevention practice that could be effectively prevent the disease transmission from animals (including meat/carcass) to human and also ask them how much they are confidence in disease prevention by practicing these measures. In addition, we also assessed the influence of unidentified factors from Model 1 on self-efficacy by taking into account the residuals from the first model (Fig. 2).

#### Descriptive statistical analysis

The data were analysed by cross-tabulation and descriptive analysis. Confidence intervals, standard errors, proportion and *p*-value were provided. Even though the outcome variables (i.e. perceived zoonoses threat and self-efficacy) were originally categorized into three: “Yes”, “No” and “Unsure”, the final outcome was categorised into only two categories which are “Yes” and “No”, with “No” being the combination of the two categories “No” and “Unsure”.

#### Social network analysis of livestock movements

Firstly, data on livestock trade connectivity between VCAs (i.e. farmers and supply chain actors) and locations of trade was collected from a total of 676 respondents. For the data analysis, two separated 2-mode networks each, for farmers-location network and supply chain actors-location network, were created by using social network analysis (SNA) to visualize the links and relationships (ties) between VCAs (nodes) of interest [34, 35]. Graph theory in SNA was used to estimate the connectivity between trading locations and each VCAs [36]. Second, to understand the livestock market chain via the VCAs in the CDZ of Myanmar, we created the 1-mode location-location network by identifying the network of trading location via VCAs.

In this study, we hypothesized that the higher connectivity in the livestock trade may contribute to information flow on zoonoses from different sources which in turn would lead to promote more awareness of VCAs on zoonoses threats. For the analysis, k-core of VCAs in livestock trading network were used as independent variables to examine the impact of connectivity on perception and awareness of VCAs on zoonoses risk and disease prevention practice. In addition, the trading locations that connected to highly connected subgroup trading locations were also identified in this study. K-core of location nodes were investigated to understand the location specific information in trading. The value of k-core in this study explained that the quantity of networks of each node in the subgroup is adjacent to the other nodes in the subgroup, thereby identifying the most influential nodes [37, 38]. The value of k-core in this study described the quantity of network of nodes (VCAs) adjacent to (i.e. traded with) each node (each VCAs) in livestock trading. Furthermore, livestock trading network mapping was also developed by using social network information from geographical livestock trading network connectivity. The software Ucinet 6 and Netdraw were used in all analyses.

#### Two stages modelling approach

Two stages multilevel mixed-effects generalized linear model was developed to identify factors associated with two dependent variables and predictors based on hypothesized causal diagram (Fig. 2). In the first stage, “perceived threat of zoonoses” was set as dependent variables and factors associated with perceived threat of zoonoses was identified. In the second stage, factors associated with the confidence in ability of VCAs to prevent zoonotic disease transmission from their animals were observed. For second stage, we used the residuals extracted from the first model (i.e. perceived threat model) as a fixed effect for association with self-efficacy for prevention of the disease. To identify the missing effect of factors not included in our model, we used the residuals extracted from the first model, which represented the factors not included in the model. Using the residuals from the first model allowed us to identify whether factors not included in the first model (i.e. residual) showed significant effect on self-efficacy [39].

#### Modelling perceived threat of zoonoses

In the first stage, multilevel mixed-effects generalized linear model was developed to identify factors associated with perceived threat of zoonoses, i.e. the knowledge of farmers on the risk and the threat of zoonoses transmitted from livestock species. Initially, we estimated the intraclass correlation coefficient (ICC) to identify whether the clustering effect of village needs to be considered for further analyses. Theoretically, ICC should be the value of > 0.05 for representing the individuals within the groups resembling each other. From the results from ICC, the perceived threat of cattle, poultry and self-efficacy in prevention of disease transmitted from cattle and small ruminants was greater than 0.05. Even though the rest of the dependent variables for this study (i.e. perceived threat of small ruminants, self-efficacy on prevention of diseases transmitted through poultry) were less than 0.05, we account villages as a random affect to be constant across all models. In the mixed linear model, response variables were fixed as family ‘binomial’ and set ‘logit’ as link function. The perceived threat was set as the dependent variable and the factors such as demographic information (e.g. age, gender, experience), k-core of livestock trading (see estimation procedures below), perceived severity, cue to action and village size were set as independent variables by accounting the random effect of village in the data analysis.

#### Modelling self-efficacy for zoonotic disease prevention

In the second stage, multilevel mixed-effects generalized linear modelling approach was conducted to identify the factors associated with the confidence in ability of VCAs to prevent zoonotic disease transmission from their animals. In the mixed linear model, response variables were fixed as family ‘binomial’ and set ‘logit’ as link function. The self-efficacy was set as dependent variables and the factors such as preventive measures, perceived barriers and residuals from first models were set as independent variables by accounting the random effect of village in the data analysis (Fig. 2).

The data was entered into a Microsoft Excel 2013 spreadsheet. Using Stata 14.0 (Stata Statistical Software, College Station, Stata Corporation, 2015), we used the survey-analysis approaches accounting for sampling weights, variance estimation (VCE), strata set up clustering effect (“Townships” as strata for primary sampling units PSUs (i.e. villages) and “Villages” as strata for secondary sampling units SSUs (i.e. households) [40–43].

## Results

### Demographic information of VCAs

The questionnaire interview was conducted to a total of 613 farmers and 63 supply chain actors in the study areas of CDZ. Of all the respondents, the proportion of female and male was not much different in farmer groups while the proportion of gender seemed to be quite different in supply chain actor group (Chi-square = 16.8, *p* < 0.001) (Table 2) with the median age of 46. A similar situation was also seen between farmer groups and supply chain actors groups (*p* < 0.05) in duration of rearing/trading cattle, goat, village chickens and type of livestock species reared or traded (Table 2). More than half of the cattle and village chicken farmers had more than 5-years experience of rearing while the majority of small ruminant farmers had less than 5-years experience. The majority of the supply chain actors across all different livestock species had more than 5-years experience. For the ownership groups of farmers, the proportion of farmers across all different groups was quite parallel. The majority of supply chain actors in this study practised village chicken trading (45.2% of total supply chain actors in this study) followed by cattle trading (29%), small ruminant trading (23%). Interestingly, trading small ruminants along with village chickens by a small proportion of supply chain actors (3.2%) is also noted. Regarding the interconnection in trading of two different groups (i.e. farmers and supply chain actors), the highly significance between the two groups was noted (Chi-square = 336.3, *p* < 0.001) (Table 2).

**Table 2:** Characteristics of livestock stakeholders (farmers and supply chain actors) in the CDZ of Myanmar (*p < 0.05; **p < 0.01; **p < 0.001)

Name of variables	Categories	Farmers		Supply chain actors		Ӽ2
		N	Proportion with 95% CI	N	Proportion with 95% CI	
Gender	Male	613	49.8 (44.2–55.4)	63	76.2 (63.8–85.3)	16.8***
	Female		50.2 (44.6–55.9)		23.8 (14.7–36.2)	
Age	≤46 years old	613	48.2 (44.2–52.2)	63	71.4 (59.0–81.3)	12.3***
	> 46 years old		51.8 (47.8–55.8)		28.6 (18.7–41.0)	
Experience of rearing/trading cattle	≤5 years	382	9.2 (6.4–13.2)	17	47.1 (24.9–70.4)	25.2***
	> 5 years		90.8 (86.8–93.6)		52.9 (29.6–75.1)	
Experience of rearing/trading sheep	≤5 years	303	87.2 (77.9–92.9)	16	25.0 (9.4–51.9)	4.5
	> 5 years		12.8 (7.1–22.1)		75.0 (48.1–90.7)	
Experience of rearing/trading goat	≤5 years	303	51.2 (43.1–59.2)	16	25.0 (9.4–51.9)	35.7***
	> 5 years		48.8 (40.8–56.9)		75.0 (48.1–90.7)	
Experience of rearing/trading chicken	≤5 years	327	23.9 (17.8–31.2)	30	16.7 (7.0–34.8)	0.7
	> 5 years		76.1 (68.8–82.2)		83.3 (65.2–93.0)	
Type of animal reared	Cattle only	613	21.0 (16.9–25.9)	63	29.0 (18.9–41.8)	77.0***
	Small ruminants only		15.9 (11.8–21.1)		22.6 (13.7–35.0)	
	Village chickens only		11.4 (8.1–15.9)		45.2 (33.0–57.9)	
	Cattle + Small ruminants		9.3 (6.0–14.1)		0	
	Cattle + Village chicken		17.8 (12.9–24.0)		0	
	Small ruminants + Village chickens		10.7 (7.7–14.7)		3.2 (0.8–12.4)	
	Cattle + Small ruminants + Village chickens		13.9 (9.8–19.3)		0	
k-core	0	613	19.4 (16.5–22.8)	63	0	336.3***
	1		75.4 (71.8–78.6)		27.0 (17.4–39.3)	
	2		5.2 (3.7–7.3)		31.8 (21.4–44.3)	
	3		0		41.3 (29.7–53.8)	

### Social network of VCAs on livestock trading

**Fig. 3:**
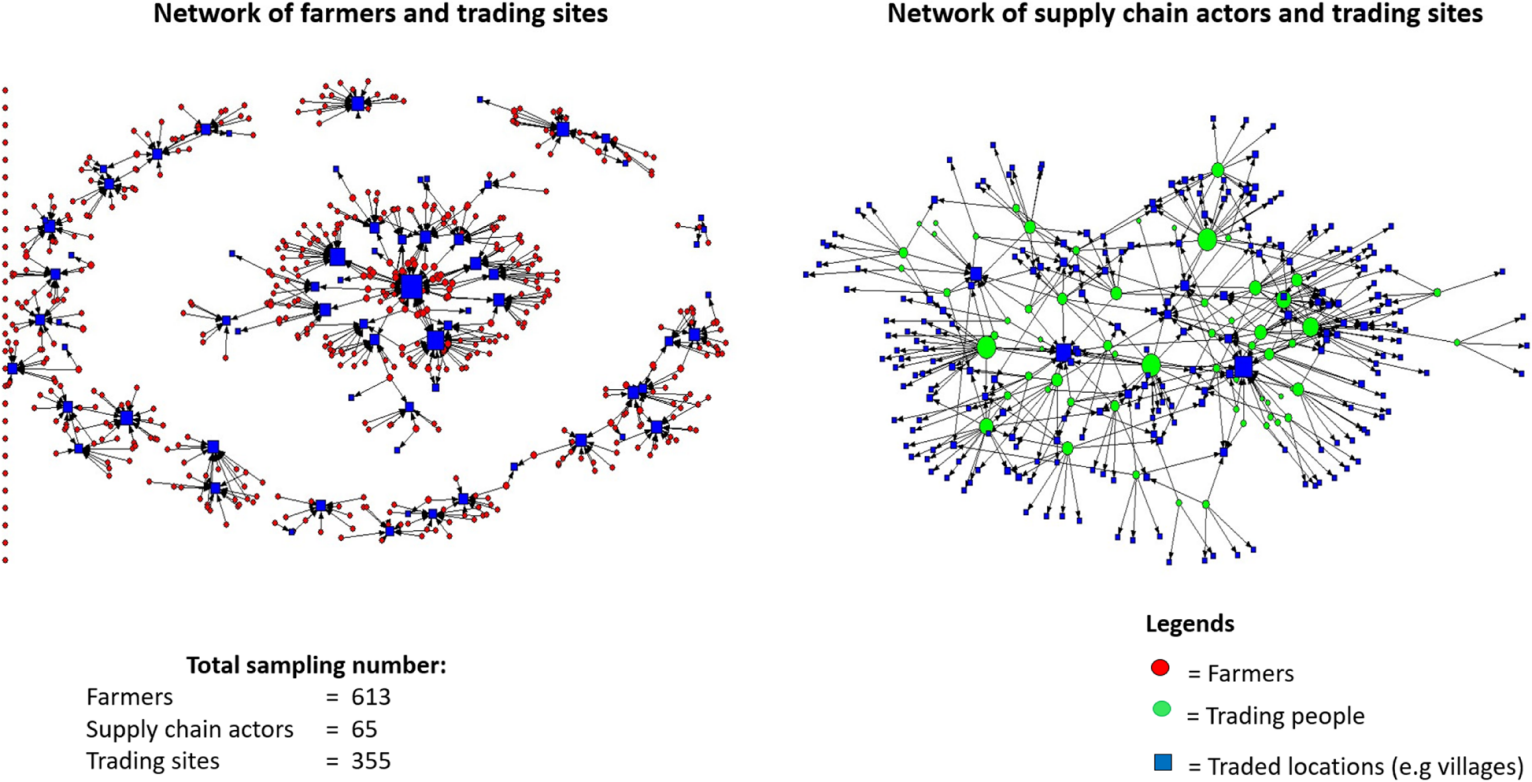
Visual social networking of livestock trading among value chain actors (i.e. farmers and supply chain actors) and the trading sites

**Fig. 4:**
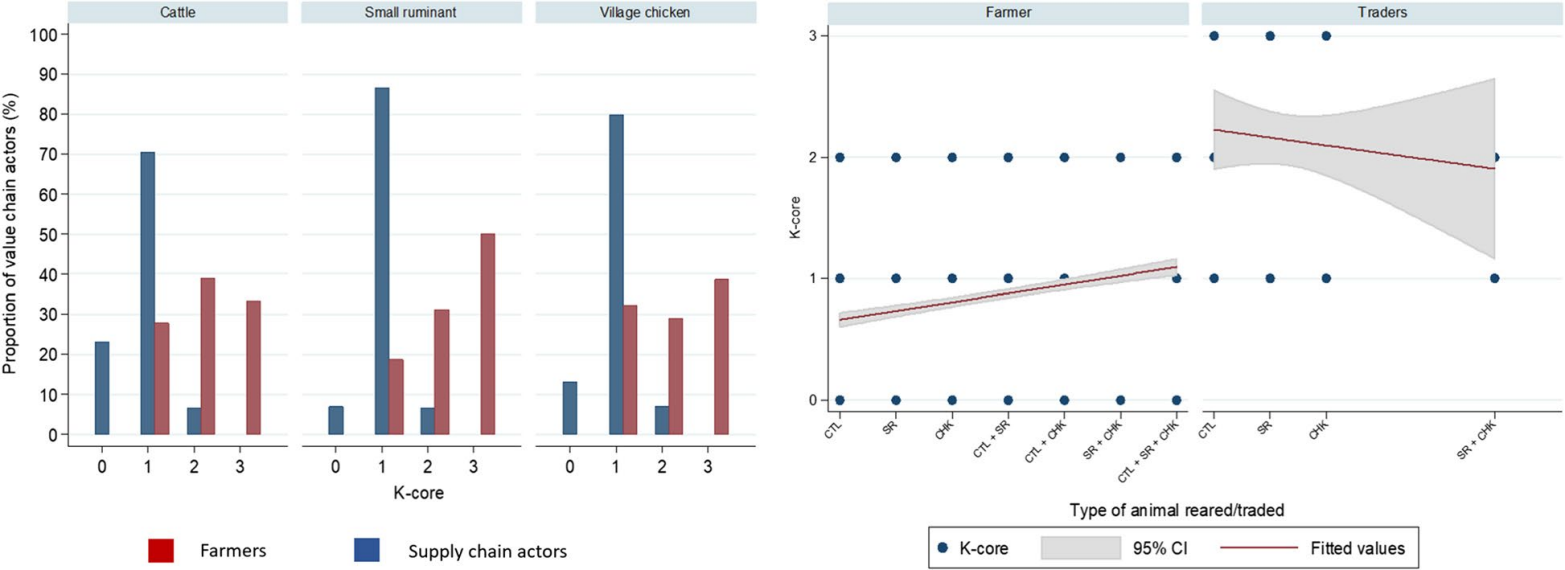
Distribution of K core for the VCAs of livestock trading in the CDZ of Myanmar indicating CTL = cattle; SR = Small ruminant; CHK = village chicken

The K-core of the livestock farmers ranged from 0 to 2 whereas the trading connectivity of supply chain actors (K-core) was ranging from 1 to 3. Our result also showed that the higher K-core was seen in the livestock supply chain actors whereas the majority of farmers had K-core of ‘zero’ which means they do not belong to a highly connected subgroup. The network showing the connectivity between farmers and trading sites is highly fragmented compared to supply chain actors. It is interesting to see that the social networking link among farmers comprised of many components. The largest giant weak component (i.e. the largest component/cluster in which each node is connected to the component by at least one direction, which mean each VCA is connected to the location by trade-in or trade-out but not both) included 201 farmer nodes and 29 location nodes, the second largest components included 72 farmer nodes and 11 location nodes, and many small components (1–22 nodes in each components). However, for the supply chain actors social network connectivity, the supply chain actors seem to practise common trading location by finding only one giant weak component composed of 63 supply chain actor nodes and 220 location nodes in total from our results (Figs. 3 and 4).

**Fig. 5:**
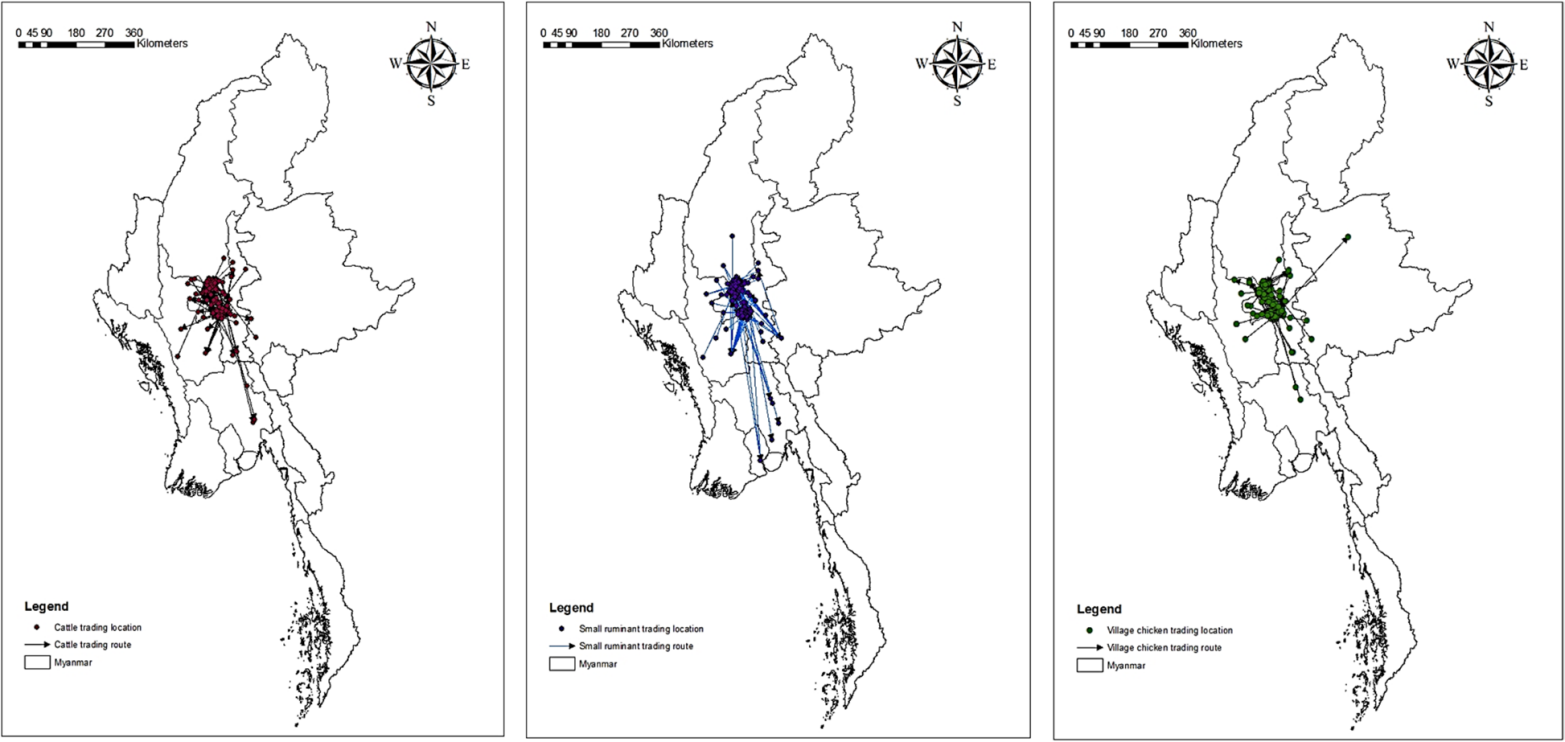
Geographical distributions of trading networks of different livestock species (cattle, small ruminant and village chicken) in the CDZ of Myanmar

Our study highlighted that livestock trade is practised not only within townships of the study areas but also outside of the study townships (Fig. 5). Among the total of 355 trading sites included in this study, a total of 59 trading sites (i.e. towns and villages) subgroup belonged to the highly connected subgroup (k-core = 4–5) (Table 3).

**Table 3:** The list of locations (i.e. villages/towns) belonging to the highest k-core (i.e. k-core = 4–5) in livestock trading network

Townships	Town/Village tract	Villages	k-core			
			Cattle trading	Small ruminant trading	Village chicken trading	All trading
Myingyan	Pyawt	Chin Myint Kyin	1	3	3	5
Myingyan	Ka Taw	Ka Taw	2	3	3	5
Meikhtila	Kan Ni	Kan Ni	1	3	3	5
Myingyan	Kyar Taing	Kyauk Kone	2	3	2	5
Mandalay	Mandalay	Cattle Market	2	2	3	5
Myingyan	Myingyan	Cattle Market	2	3	3	5
Myingyan	Nwar Ku Aing	Nwar Ku Aing	2	3	3	5
Myingyan	Hpet Pin Aing	Hpet Pin Aing	2	3	3	5
Myingyan	Yathar	Phat Yin	0	3	3	5
Myingyan	Pin Lel	Pin Lel	1	3	3	5
Myingyan	Si Mee Khon	Si Mee Khon	2	3	3	5
Myingyan	Taw Pu	Taw Pu	2	3	3	5
Myingyan	Yathar	Yathar	2	2	3	5
Meikhtila	Ah Lel	Ah Lel	2	2	1	4
Meikhtila	Shwe Sit Thi	Aung Thar	2	2	1	4
Mahlaing	Hpyauk Seik Kone	Hpyauk Seik Kone	0	3	1	4
Meikhtila	Kyaut Phoo	Hta Naung Kone	1	3	2	4
Myingyan	Hta Naung Taing	Hta Naung Taing	2	3	2	4
Meikhtila	Tha Yet Pin	Aint Kone	2	1	2	4
Meikhtila	Sat Pyar Kyin	Kan Gyi Kone	0	3	3	4
Meikhtila	Lein Taw	Kan Kaung	2	1	2	4
Meikhtila	Yae Wai	Kan Thar	1	2	3	4
Natogyi	Khat Lan	Khat Lan	1	3	2	4
Meikhtila	Thee Pin Kone	Kone Tan	2	3	1	4
Meikhtila	Gway Aing	Kwae Tauk Kan	2	3	3	4
Meikhtila	Kyauk Hpu	Kyauk Hpu	2	1	1	4
Meikhtila	Nyaung Pin Sho	Kyauk Pone	2	1	0	4
Meikhtila	Tha Yet Pin	Kyee Thar Aik	0	3	1	4
Meikhtila	Thee Pin Kone	Kyi Kone	2	3	3	4
Myingyan	Gyoke Pin	Gyoke Pin	2	3	3	4
Kyaukpadaung	Let Pan Pyar	Let Pan Pyar	0	0	3	4
Ma Hlaing	Ma Hlaing	Cattle Market	2	2	2	4
Meikhtila	Meikhtila	Cattle Market	2	3	3	4
Myingyan	Thar Paung	Myauk Kyone	2	3	1	4
Myingyan	Pyawt	Myin Thar	2	2	3	4
Ngazun	Myo Thar	Myo Thar	2	1	1	4
Meikhtila	Myauk Lel	Myauk Lel	1	2	3	4
Myingyan	Thin Pyun	Nyaung Pin Thar	2	3	2	4
Myingyan	Nyaung Wun	Nyaung Wun	2	1	1	4
Meikhtila	Mway	Oh Ma Twayt	2	3	2	4
Meikhtila	Ohn Ton	Ohn Ton	2	3	2	4
Ngazun	Pauk Sein	Pauk Sein	0	0	3	4
Meikhtila	Sat Pyar Kyin	Sat Pyar Kyin	2	3	3	4
Meikhtila	Shaw Hpyu Kan	Shaw Hpyu Kan	2	3	2	4
Meikhtila	Za Yat Kone	Hlyaw Hpyu Pin	2	3	3	4
Myingyan	Pyawt	Shwe Pone Thar	2	1	3	4
Kyaukpadaung	Taung U	Taung U	0	0	3	4
Meikhtila	Taw Ma	Taw Ma	2	0	0	4
Meikhtila	Sat Pyar Kyin	Tha Hpan Pin Yoe	1	2	3	4
Meikhtila	Mon Taing	Tha Yet Chan	2	3	3	4
Meikhtila	Tha Yet Pin	Tha Yet Pin	2	3	3	4
Meikhtila	Myauk Lel	Tha Yet Tan	2	1	2	4
Taungtha	Wea Laung	Wea Laung	1	3	2	4
Meikhtila	Taw Ma	Yae Cho	1	3	3	4
Meikhtila	Myauk Lel	Yae Ngan (West)	2	2	3	4
Meikhtila	Yae Wai	Yae Wai	2	3	3	4
Meikhtila	Yae Cho	Ywar Thar	0	3	3	4
Meikhtila	Yae Wai	Ywar Thit	1	3	2	4
Meikhtila	Za Yat Kone	Za Yat Kone	2	1	3	4

### Perception of VCAs on zoonoses

Table 4 presents the results obtained from the analysis of the perceptions of the farmers and supply chain actors on risks from animal species for zoonotic disease transmission. From the data, it was seen that a greater proportion of supply chain actors thought cattle posed a moderate or high zoonotic disease risk than farmers, with the majority of the latter believing that cattle posed no zoonotic risk (*p* < 0.05). In addition to this, we found a significant difference between supply chain actors and farmers in the perception of level of zoonoses severity risk across different livestock species (*p* < 0.001).

**Table 4:** Health belief criteria of VCAs on the zoonotic diseases (*p < 0.05; **p < 0.01; **p < 0.001, ^a^ = % of a total survey population)

Health belief criteria	Questions	Species	Categories	Farmers (%) ***N*** = 613	Supply chain actors (%) ***N*** = 63	Ӽ2
Perceived threat	Which species of animal do you think can transmit zoonotic disease to human?	Cattle	Yes	16.6 (13.9–19.8)	49.2 (36.8–61.7)	38.3***
			No	83.4 (80.2–86.1)	50.8 (38.3–63.2)	
		Small ruminant	Yes	9.1 (7.1–11.7)	9.5 (4.3–19.8)	0.01
			No	90.9 (88.3–92.9)	90.5 (80.3–95.7)	
		Poultry	Yes	48.3 (44.3–52.3)	65.1 (52.3–76.0)	6.4*
			No	51.7 (47.7–55.7)	34.9 (24.0–47.8)	
Perceived severity	Which level do you consider the impacts of the risk of transmissible diseases from animal to human on human health?	Cattle	None	83.4 (80.2–86.1)	50.8 (38.3–63.2)	126.3***
			Moderate	2.1 (1.2–3.6)	34.9 (24.0–47.8)	
			High	14.5 (11.9–17.5)	14.3 (7.5–25.6)	
		Small ruminant	None	94.9 (92.9–96.4)	90.5 (80.0–95.8)	16.0***
			Moderate	0.7 (0.3–1.7)	6.3 (2.3–16.1)	
			High	4.4 (3.0–6.4)	3.2 (0.8–12.2)	
		Poultry	None	48.3 (44.3–52.3)	65.1 (52.5–75.9)	17.7***
			Moderate	9.8 (7.7–12.4)	19.1 (11.1–30.8)	
			High	41.9 (38.1–45.9)	15.9 (8.7–27.2)	
Perceived barrier	What are the barriers for preventive measures?	Cattle	No barrier	82.9 (79.7–85.7)	98.4 (89.1–99.8)	10.5*
			Financial constraint	2.8 (1.7–4.4)	0	
			Limited knowledge	9.0 (6.9–11.5)	1.6 (0.2–10.9)	
			Limited resource	5.4 (3.9–7.5)	0	
		Small ruminant	No barrier	89.6 (86.9–91.7)	98.4 (89.1–99.8)	5.4
			Financial constraint	2.1 (1.2–3.6)	0	
			Limited knowledge	4.6 (3.2–6.5)	1.6 (0.2–10.9)	
			Limited resource	3.8 (2.5–5.6)	0	
		Poultry	No barrier	89.1 (86.3–91.3)	98.4 (89.1–99.8)	5.6
			Financial constraint	2.0 (1.1–3.4)	0	
			Limited knowledge	6.5 (4.8–8.8)	1.6 (0.2–10.9)	
			Limited resource	2.5 (1.5–4.0)	0	
Cue to action	How do you obtain the information to prevent disease transmission from animal to human?	Cattle	No information obtained	54.2 (50.2–58.1)	88.9 (78.1–94.7)	51.0***
			Other farmers	21.0 (18.0–24.5)	3.2 (0.8–12.2)	
			Media	3.1 (2.0–4.8)	0	
			Local authorities	21.7 (18.6–25.2)	4.8 (1.5–14.1)	
			Other traders	0	3.2 (0.8–12.2)	
		Small ruminants	No information obtained	72.9 (69.3–76.3)	88.9 (78.1–94.7)	29.9***
			Other farmers	13.5 (11.1–16.5)	3.2 (0.8–12.2)	
			Media	1.8 (1.0–3.2)	0	
			Local authorities	4.8 (1.5–13.9)	4.8 (1.5–14.1)	
			Other traders	0	3.2 (0.8–12.2)	
		Poultry	No information obtained	74.2 (70.6–77.5)	88.9 (78.1–94.7)	28.0***
			Other farmers	10.1 (8.0–12.8)	3.2 (0.8–12.2)	
			Media	2.9 (1.9–4.6)	0	
			Local authorities	12.7 (10.3–15.6)	4.8 (1.5–14.1)	
			Other traders	0	3.2 (0.8–12.2)	
Self-efficacy	Do you think you can prevent the disease being transmitted from animal to human?	Cattle	Yes ^a^	53.3 (49.4–57.3)	55.2 (41.9–67.7)	1.2
		Small ruminants	Yes ^a^	37.7 (33.9–41.6)	55.2 (41.9–67.7)	1.7
		Poultry	Yes ^a^	41.1 (37.3–45.1)	55.2 (41.9–67.7)	0.6

Overall, VCAs who responded for preventive measures highlighted practising a number of preventive measures including burying the suddenly dead animals, not eating contaminated meat, treating their own sick animals and keeping their animals away from humans. On the other hand, it was interesting to see that the majority of SCAs (> 85%) reported that they did not practise any preventive measures.

The majority of the VCAs [farmers (82.9, 95%CI: 79.7–85.7) and supply chain actors (98.4, 95%CI: 89.1–99.8)] mentioned that they had no barriers to implement preventive measures. However, respondents described a number of barriers to practising disease prevention measures which included financial constraint (i.e. no funds to conduct prevention practices, not able to avoid eating infected carcass with low price due to poverty), limited knowledge (i.e. no knowledge about zoonotic diseases and how to prevent the disease being transmitted from livestock to humans) and limited resources (i.e. no separate shelter to keep livestock, limited veterinary service to treat sick animal, limited resources such as disinfection, medicine, feed containers for sanitation and poor biosecurity practices). Limited knowledge of preventive measures stood out as the most common problem across VCAs: farmers (9.0, 95%CI: 6.9–11.5) and supply chain actors (1.6, 95%CI: 0.2–10.9). Interestingly, it was seen that the barriers which occurred across different cattle VCAs were significantly different (Chi-square = 10.5; *p* < 0.05) while there is no difference across different stakeholder groups of other livestock species (Table 4).

Respondents from this study reported a number of sources of information for the awareness of the risk of zoonoses and prevention measures which are the farmers, media and local authorities. 54, 73 and 74% of cattle, small ruminant and village chicken farmers and 89% each of cattle, small ruminant and village chicken supply chain actors, respectively, reported they had obtained no information about zoonotic disease prevention from any source. On the other hand, it was noted that the main sources for public awareness of zoonoses risk were local authorities and farmers across different livestock species groups while the role of the media in public awareness was low (< 5%). However, the availability of knowledge on zoonoses was different between farmers and supply chain actors indicating from the data that showed that a higher proportion of farmers reported the availability of knowledge than trader groups (*p* < 0.001). In addition, our findings indicate that the source of information for zoonoses prevention was significantly different across livestock stakeholders (*p* < 0.001) (Table 4).

Regarding the disease prevention practices, the majority of farmers practiced the “not eating the carcass of infected cattle” (45.7% (41.8–49.7)) while the practice of treating sick animals seem to be fairly distributed in the prevention of zoonotic diseases from cattle, small ruminant and village chicken (Table 5). SCAs did not aware of the effectiveness of prevention or control methods for specific livestock diseases and they reported that “the way they practiced could prevent all the zoonotic diseases”. Among these, the proportion of SCAs practicing hand hygiene was fairly highly (9.5, 95%CI: 4.3–20.0)) compared to other practices such as bury dead animals, quarantine the sick animals, and cooking the meat well (4.8, 95%CI: 1.5–14.1) (Table 6).

**Table 5:** Zoonotic disease prevention measures practiced by farmers raising different livestock species (*N* = 613)

Prevention measures	Preventive measures for zoonotic disease transmitted from:		
	Cattle (%)	Small ruminant (%)	Poultry (%)
Bury dead animal	18.6 (15.7–21.9)	0.2 (0.02–1.2)	6.9 (5.1–9.2)
Not eating the carcass of infected animal	45.7 (41.8–49.7)	3.1 (2.0–4.8)	7.0 (5.2–9.3)
Hand hygiene	10.9 (8.7–13.7)	16.8 (14.0–20.0)	15.2 (12.5–18.2)
Treating sick animal	16.8 (14.0–20.0)	15.7 (13.0–18.8)	7.8 (5.9–10.3)
Quarantine the sick animal	21.7 (18.6–25.2)	3.1 (2.0–4.8)	3.1 (2.0–4.8)

**Table 6:** Zoonotic disease prevention measures practiced by supply chain actors (*N* = 63)

Prevention measures	Supply chain actors (%)
Bury dead animal	4.8 (1.5–14.1)
Hand hygiene	9.5 (4.3–20.0)
Quarantine the sick animal	4.8 (1.5–14.1)
Cooking the meat well	4.8 (1.5–14.1)

### Factors affecting the perceived threat on zoonoses by livestock VCAs

In our first model we examined factors including demographic information, perceived severity, cue to action, associated with the perceived zoonoses threat transmitted from three livestock species (i.e. cattle, small ruminants and poultry) (Table 7). After initial descriptive analysis the variable Perceived Severity was excluded from further analysis due to the fact that there was no variation in responses between VCAs. Perceived threat differed between the gender of VCAs, with males 1.5 times more likely to be aware of the threat of zoonoses transmitted from cattle and poultry than females (*p* < 0.05). Furthermore, the type of VCAs was also associated with the perceived threat of zoonoses by different livestock species. More supply chain actors than farmers were aware of zoonoses transmitted by cattle (*p* < 0.05) while farmers not working with small ruminants and poultry were less likely to be aware of the risk of zoonoses from these animals than farmers working with these livestock species. Our results also indicate that the availability of information on zoonoses was associated with perceived threat of zoonoses. Farmers were the major source that promoted the awareness of VCAs on zoonoses transmitted from small ruminants (OR = 2.2, *p* < 0.05). However, the awareness of VCAs on zoonoses transmitted from poultry was promoted by three different sources of information (i.e. media: OR = 5.4, *p* < 0.01; other farmers: OR = 2.0, *p* < 0.05; local authorities: OR = 2.5, *p* < 0.01) (Table 7).

**Table 7:** Final multilevel mixed effect generalized binomial linear modelling with a random effect of location (villages) to understand the factors affecting perceived threat of VCAs on zoonotic diseases transmission

Variables		Perceived threat of risk animal (Odds ratio)		
		Zoonosis from cattle	Zoonosis from small ruminant	Zoonosis from poultry
***Modifying factors***				
Age (Ref: ≤46 y.o Vs > 46 y.o)		1.0 (0.6–1.5)	1.4 (0.8–2.4)	1.0 (0.7–1.5)
Gender (Ref: Female Vs Male)		1.5* (1.0–2.3)	1.2 (0.7–2.1)	1.5* (1.1–2.2)
Experience of rearing/trading: (Ref: ≤5 years)	Cattle (> 5 years)	0.8 (0.4–1.4)	0.8 (0.4–1.4)	1.0 (0.7–1.5)
	Sheep (> 5 years)	0.6 (0.3–1.3)	0.9 (0.4–2.2)	1.0 (0.6–1.9)
	Goat (> 5 years)	1.0 (0.6–1.7)	1.2 (0.6–2.3)	1.1 (0.7–1.6)
	Poultry (> 5 years)	1.4 (0.9–2.3)	1.9 (1.0–3.6)	0.8 (0.5–1.2)
Trading connectivity (Ref: K-core 2–3)	K-core 1	1.0 (0.5–2.2)	3.1 (0.8–11.5)	0.7 (0.4–1.5)
	K-core 0	1.4 (0.6–3.4)	2.9 (0.6–14.1)	0.8 (0.4–1.9)
Type of VCAs (Ref: F1)	F2	0.6 (0.3–1.0)	0.3** (0.1–0.7)	0.5** (0.3–0.7)
	T1	4.3* (1.2–15.5)	2.0 (0.3–13.4)	0.5 (0.1–1.6)
	T2	5.6** (1.9–16.7)	1.0 (0.2–1.9)	0.1** (0.03–0.4)
Cue to action (Ref: None)	Other farmers	1.3 (0.7–2.3)	2.2* (1.1–4.5)	2.0* (1.1–3.6)
	Media	0.6 (0.1–2.6)	0.8 (0.1–6.8)	5.4** (1.4–20.5)
	Local authorities	1.2 (0.7–2.2)	1.3 (0.5–2.9)	2.5** (1.4–4.4)
	Other supply chain actors	1.0	10.7 (0.4–282.9)	5.4 (0.2–143.6)
Overall p-value of the model		0.0004	0.0019	0.0000
Intercepts		0.17	0.03	1.49
Likelihood ratio		0.19	0.01	17.45

*F1* Farmers raised specific species (cattle, small ruminant or village chicken), *F2* Farmers did not raise specific species, T1 = Supply chain actors traded specific species (cattle, small ruminant or village chicken); T2 = Supply chain actors did not trade specific species

*p < 0.05; **p < 0.01; ***p < 0.001

### Factors affecting self-efficacy on zoonoses by livestock VCAs

Our second model examined the factors influencing the self-efficacy of farmers for zoonoses prevention across different livestock species, including preventive practices for zoonoses transmitted from livestock (i.e. bury dead animals, not eating the carcass of infected animals, hand hygiene, treating sick animal, quarantine the sick animal), perceived barriers (i.e. financial constraints, limited knowledge, limited resources), and residual from the first model (i.e. the unidentified factors on perceived threat). From our model, the VCAs who would not eat meat from sick cattle were less likely to report that they were confidence managing zoonotic disease risk. Amongst VCAs working with small ruminants, other prevention practices such as zoonoses prevention practice of proper hand hygiene (i.e. cleansing the hand properly after touching, cutting, cooking the meat) and treating the sick animal were positively associated with confidence in prevention of zoonoses transmission (*p* < 0.05). The residuals from the first models seems to be highly significant among different livestock species which showed that the unidentified variables in the first model seems to affect the confidence in ability of zoonosis prevention of VCAs on zoonotic diseases transmission. Similarly, reported prevention practice of treating sick chickens was also positively associated with the self-efficacy of VCAs on prevention. Similar to self-efficacy on preventing transmissible zoonoses from cattle, limited knowledge was observed as the main factor negatively associated with the self-efficacy of preventing transmissible zoonoses from small ruminants. However, the other factors such as perceived barriers were not significantly different in self-efficacy on prevention of zoonoses transmitted from poultry (Table 8).

**Table 8:** Final multilevel mixed effect generalized binomial linear modelling with a random effect of location (villages) to understand the factors affecting confidence in ability of zoonosis prevention of VCAs on zoonotic diseases transmission

Variable		Confidence in ability of zoonosis prevention (Odds ratio)		
		Zoonosis from cattle	Zoonosis from small ruminant	Zoonosis from poultry
***Preventive measures***				
Bury death animal (Ref: No Vs Yes)		1.0 (0.4–2.3)	1.0	0.7 (0.1–5.6)
Not eating the carcass of infected animal (Ref: No Vs Yes)		0.2*** (0.1–0.4)	2.2 (0.7–3.7)	2.0 (0.2–17.0)
Hand hygiene (Ref: No Vs Yes)		1.9 (0.6–5.6)	7.7*** (4.1–14.3)	1.6 (1.0–2.7)
Treating sick animal (Ref: No Vs Yes)		1.7 (0.6–4.5)	7.3*** (3.8–13.9)	2.2* (1.1–4.6)
Quarantine the sick animal (Ref: No Vs Yes)		1.0 (0.4–2.9)	2.2 (0.7–7.1)	2.7 (0.9–8.2)
Residuals from the first model (From first model: perceived threat)		414.8** (13.9–12,416.1)	3039838*** (63,199.3–1.46e+ 08)	175.1*** (34.3–893.8)
Perceived barrier (Ref: None)	Financial constraint	1.2 (0.4–4.4)	2.9 (0.6–13.7)	2.3 (0.6–8.8)
	Limited knowledge	0.3*** (0.2–0.6)	0.4* (0.1–1.0)	0.5 (0.2–1.0)
	Limit resources	0.4* (0.2–1.0)	0.8 (0.3–2.7)	0.8 (0.2–2.7)
Overall p-value		0.0000	0.0000	0.0000
Intercepts		0.99	0.07	0.04
Likelihood ratio test		0.14	5.59	3.35

*p < 0.05; **p < 0.01; ***p < 0.001

## Discussion

In this study we compared perceptions and practices between farmers and livestock supply chain actors in the CDZ with respect to zoonotic risks and investigated the factors associated with perceived threat and self-efficacy practices towards zoonotic risks from their livestock. The factors identified in this study can help support the development of disease prevention and health promotion strategies to enhance the health of farmers and supply chain actors under the One-Health paradigm in the CDZ of Myanmar.

Animal movement and trade has been highlighted as an important factor for disease spread [24, 25, 44, 45]. The interaction of farmers and supply chain actors through these livestock trade channels could potentially also contribute to the dissemination of information on disease prevention and control. Our results from the social network of livestock movement in the CDZ of Myanmar demonstrate that the livestock trading network in the CDZ is complex and different between stakeholders involved in the livestock trading network. Not surprisingly, our results indicate that the network of livestock movements was significantly more fragmented in the farmer group compared to the trader group. The majority of cattle and village chicken farmers had K-core = 0 which did not belong to the highly connected groups whereas the majority of small ruminant farmers (K-core = 1–2) and supply chain actors (K-core = 2–3) showed their contribution in highly connected groups of livestock trading. While supply chain actors of small ruminants also often traded village chicken, the connectivity of these supply chain actors was lower compared to supply chain actors who traded single species. This might be due to cattle farmers in CDZ raising cattle mainly for draught purpose (Chapter 4) and keeping cattle for longer compared to small ruminants. Even though the literature from Myanmar supporting this finding is not available, another possible reason might be the instability of market price, market demand, accessibility of market or traders, banning due to outbreak, and disease affecting livestock trading [9, 10, 46–48] Due to the high livestock density in CDZ, the livestock were widely traded from CDZ to other parts of the country and CDZ could be one of the potential areas for disease spread. Therefore, for the control of disease spread, promoting the awareness of the nodes (i.e. supply chain actors and locations) is of paramount importance for the control of regional zoonotic diseases spread through trading.

Previous studies indicated that social background of people (i.e. income, education, religion, race or ethnicity, region, and gender) influences beliefs and perception in many aspects [49–51]. Our results also highlight that social status and occupation are important determinants of the perceived threat of zoonosis for each livestock species. Similar to other studies from developing countries, our study also supports the idea of gender playing a considerable role in the awareness of zoonosis and the perception of risk for different livestock species [52–54] in that males were 1.5 times more likely to be aware of zoonotic threat than females (*p* < 0.05). The observed gender differences may be explained by difference in limiting factors for information access such as education and social status, and further studies are needed to investigate this in more detail. Since Asian countries have been loudly alerted by the threat of Highly Pathogenic Avian Influenza [55], the campaign on transmissible zoonoses from avian species seems to have successfully promoted the awareness of VCAs on the disease threat, with a greater proportion of farmers reporting a perceived disease threat from poultry than other livestock species examined in this study. However, the differences in threats perceived between different animal species was less consistent amongst supply chain actors, with a greater proportion perceiving threats from cattle or village poultry than from small ruminants. Another finding from our study highlighted that the VCAs not working with village chicken had less awareness of the zoonoses transmitted from poultry. This finding is consistent for farmers not raising small ruminant, who were less aware of the zoonosis transmitted from small ruminant. Except for cattle diseases, the type of career seems to influence the perception of zoonosis threat transmitted from cattle. Supply chain actors, regardless of the livestock species they were working with, were aware of zoonosis from cattle and poultry which may be due to public awareness campaign of veterinary authorities on anthrax and avian influenza (Personal communication with Dr. Kyaw Naing Oo and Dr. Win Myint Thein, Livestock Breeding and Veterinary Department). Other possible reasons might be gender, education, wealth, previous experience of diseases by the supply chain actors [53, 56, 57]. To explain in this case, a possible reason might be that VCAs gave more attention to the livestock species they were working with and tended to ignore the zoonotic diseases transmitted from other livestock species or the public awareness of zoonotic disease was not widely established to cover all livestock stakeholders regardless of the livestock species they are working with. The frequency of trading and communication with different stakeholders does not seem to promote VCAs’ awareness of the zoonosis risk transmitted from livestock. This might be another issue to consider which may lead to the spread of diseases by trading routes due to the lack of awareness of diseases and lack of disease prevention practices.

To investigate determinants of self-efficacy of VCAs on zoonoses prevention, we considered the contribution of perceived threat of risk from each of the species in our study, disease prevention methods and barriers. The results of our study have important implications for the development of future disease control strategies and health promotion policies. First, our findings suggested that factors unaccounted by the perceived threat model are associated with the self-efficacy of VCAs towards zoonotic disease risk from their livestock. While the role of gender showed a significant effect on the perceived threat, we lacked other social factor data to consider in this study. The possible confounding factors such as social status, education and wealth could possibly be related to gender [58, 59]. Second, it is not surprising that limited knowledge on prevention was reported as a significant constraint in reducing the VCAs’ confidence on disease prevention in this region. The high proportion of VCAs reporting lack of information available can explain this finding. Together, these findings highlight a need for the development and testing of local, national effective public awareness campaigns on zoonoses and prevention methods based on the nature of occupations, including campaigns targeting the provision of information on zoonosis risk, better farm/market biosecurity, prevention methods. In addition, the easily accessible public awareness systems (i.e. mobile/ web application, social media, zoonotic disease information hotline) should be designed. Third, our findings suggested even though public awareness on zoonoses seems to be poor due to limited availability of information in the study area which reflected the small proportion of study population practised basic zoonosis prevention methods. We hypothesized the high k-core (which means the highly connected and interact with different value chain actors) could have more awareness on the zoonoses since they travelled and interacted with different areas and different stakeholders. According to the non-significance results from our findings, we do aware that it would be urgently needed to raise the public awareness since the interaction and networking did not show significant impact on public awareness of zoonoses and disease control. What is interesting in the above results is that even though VCAs reported a number of prevention methods to prevent disease transmission from cattle, none of them seemed to significantly promote self-efficacy. Nevertheless, the prevention practice to prevent diseases transmitted from small ruminant and poultry seem to effectively promote the self-efficacy of VCAs.

A number of study limitations need to be considered to assist the interpretation of our findings. Firstly, these findings are limited by the use of cross-sectional design and are not able to identify the perception on zoonosis of the livestock stakeholders over time. Secondly, the sample was aimed to be representative of the different livestock stakeholders in the CDZ of Myanmar but for trader groups, the data collection was able to be conducted only by means of targeted and convenience sampling so that we might have missed some of the people and selection bias was unavoidable. Thirdly, even though structural models implementing causal path-like relationships of the Health Belief framework with at least four levels of perception or awareness in each component has been used for most of the Health Belief model studies [60], we used an adapted structural Health Belief framework with two levels of perception or awareness in each Health Belief component in our study. Fourth, this study was unable to identify the effect of social factors such as wealth, education and social status. Despite these shortcomings the current findings add to a growing body of literature on the perceptions of different stakeholders in the CDZ of Myanmar on zoonotic disease.

## Data Availability

The questionnaire and data-sets used and/or analysed during the current study available from the corresponding author on reasonable request.

## Abbreviations

CDZ: Central Dry Zone
UQ: The University of Queensland
FMD: Foot and Mouth Disease
ND: Newcastle Disease
HBM: Health Belief Model
ACIAR: Australian Center for International Agriculture Research
LBVD: Livestock Breeding and Veterinary Department
PSUs: Primary sampling units
SSUs: Secondary sampling units
VCE: Variance estimation
FPC: Finite population correction
mm: Millimetre
IQR: Interquartile rate
μ: Mean value
CI: Confident interval
HH: Household
AI: Artificial insemination
